# Gene and Tissue Engineering For the Treatment of Diabetes and Its Retinal Complications: The Use of Nucleic Acid Constructs Bearing A *TXNIP* Gene Promoter

**DOI:** 10.19080/CTBEB.2018.13.555869

**Published:** 2018-04-10

**Authors:** Lalit P Singh

**Affiliations:** 1 Department of Anatomy and Cell Biology, Wayne State University School of Medicine, USA; 2 Department of Ophthalmology, Wayne State University School of Medicine, USA

**Keywords:** *TXNIP* promoter, Gene therapy, Tissue engineering, Adipose stem cells, Diabetes, Diabetic retinopathy

## Abstract

Diabetes is a chronic disease in which insulin production is deficient (Type 1) or resistant (Type 2) leading to organ complications including the heart, kidney, retina, and peripheral nerves. About 10% of diabetics are Type 1 while ~90 percent are Type 2 associated with life style changes and obesity. Whether it is Type 1 or Type 2, chronic hyperglycemia prevails and associated oxidative stress and low grade inflammation are considered to play critical roles in diabetes and its complications including diabetic retinopathy (DR). Thioredoxin-Interacting Protein, TXNIP, is strongly induced by diabetes and high glucose in all tissues examined including the pancreatic beta cells and the retina. TXNIP binds to and inhibits the anti-oxidant and thiol reducing capacity of thioredoxins and causes cellular oxidative stress, inflammation and premature cell death. TXNIP is induced strongly by high glucose and its metabolites with minutes and remains elevated as long as hyperglycemia persists. Therefore, the *TXNIP* gene promoter linked with insulin or a gene of interest may be used to induce gene expression or suppression and in tissue engineering for adipose or tissue-derived autologous stem cells producing insulin for the treatment of diabetes and its complications such as DR as well as age-related neurodegenerative diseases.

## Introduction

Diabetes or diabetes mellitus is a chronic disease which affects all organs in the body. The etiology of this disease is complex but involves either insulin deficiency or insulin resistance in the body. Therefore, there are 2 main types of diabetes -Type 1 (T1D) and Type 2 (T2D). T1D is an autoimmune disease that destroys pancreatic beta cells which produce insulin [1]. T1D can begin at an early age (from 3 months old child to teenagers); therefore T1D is also known as Juvenile diabetes. There is no cure for T1D and requires insulin injection throughout the life of the patient. Insulin is required for the uptake of blood glucose after meals by muscle, liver and fat cells for storage as glycogen or fat and for later use as energy source. On the other hand, T2D is mostly associated with obesity and peripheral insulin resistance that prevents/reduces uptake of blood glucose by muscle, liver and fat even in the presence of insulin [2]. Initially, drug treatment such as metformin is needed in T2D to sensitize glucose uptake in tissues mentioned above [3]. However, at later stages of T2D, insulin injection is also needed due to beta cell death. Subsequently, uncontrolled diabetes leads to chronic complications of the kidney, eye, nerves and the heart [3,4]. The socio-economic burden of diabetes in families and societies is enormous. Currently, due to increases in the number of obese people [5], both in developed and developing countries including the US, Europe, China and India, there is an urgent need for the development of long-term effective treatment or cure for diabetes.

We and others have shown that a protein called thioredoxin-interacting protein (TXNIP), both protein and mRNA, is strongly induced by diabetes and high glucose in all tissues examined including pancreatic beta, renal and retinal cells [6-10]. TXNIP binds to and inhibits thioredoxin (Trx), an anti-oxidant and cellular thiol reducing protein. Trx1 is found in the cytosol and nucleus while Trx2 is localized in the mitochondrion. TXNIP localizes both in the cytosol and mitochondrion, therefore, its overexpression under chronic hyperglycemia causes cellular oxidative stress and premature cell death, including beta cells. We have previously shown that the TXNIP promoter exists as opened and poised configuration in cells and that high glucose and glucose metabolites activate its expression strongly and acutely [8]. Therefore, we proposed that the TXNIP promoter can be linked with an insulin gene or a gene that induces insulin expression in non-beta cells using adult stem cell engineering [9]. Although various cell types including beta cell itself have been tried for diabetes cell therapies, their survival time is short and need frequent transplant or injection [11]. Furthermore, immune rejection occurs against the transplanted cells, especially for Type 1 diabetes as it is an autoimmune disease. Conversely, adipose-derived stem cells live long and autologous ASCs can be transplanted subcutaneously safely without rejection in diabetic patients. This will eliminate daily insulin injections by both T1D and T2D patients.

## Diabetic Retinopathy

Diabetic retinopathy (DR) is the number one cause of blindness among the adult working population in the US. Because of the increase in the incidence of obesity, diabetes, and hypertension around the globe, which I termed together as Diabesithy, the number of people with DR is expected to increase significantly. DR was considered as a microvascular complication of the eye affecting the capillary endothelial cells, pericyte dropout, basement membrane thickening and leakage of the blood vessels, i.e., the breakdown of the inner blood-retinal barrier (iBRB) leading to infiltration of blood components into the vitreous and neuroretina [12,13]

The retina being a part of the central nervous system consumes large amounts of glucose and oxygen for its bioenergetics via the mitochondrial election transport chain, and is required for photo transduction and visual perception. Recently, neuroinflammation and neurodegeration the diabetic retina has been reported and is considered to occur before microvascular damages [13,14]. Therefore, DR may also be considered as a neurodegenerative disease. During bioenergetics in mitochondria and as byproducts of the oxidative phosphorylation in electron transport chains, electrons leak into the mitochondrial matrix and the mitochondrial intermembrane, which are captured by molecular oxygen to generate high reactive oxygen radicals and reactive oxygen species. Although there are antioxidant systems in retinal cells, under acute or chronic disease conditions, the reactive oxygen species accumulate and damage mitochondrial proteins, DNA and membrane lipids thereby damaging the organelle.

Damaged mitochondria are infective in ATP synthesis but continue to produce ROS. Therefore, the removal of damaged mitochondria via lysosomal degradation called mitophagy, a specific form of autophagy to remove non-functional mitochondria [14,15]. If damaged mitochondria are not removed, they release mtROS, mtDNA and lipid components, which are considered as danger molecules, termed damaged/danger-associated molecular patterns (DAMPs) therefore, a cellular innate defense response is evoked. Particularly, mitochondrial DAMPs are recognized by NOD-like receptors such as NLRP3 inflammasome and activates pro-caspase 1 to caspase 1, which further processes pro-inflammatory cytokines, pro-IL-1β and pro-IL-18, to their active forms of IL-1β and IL-18, respectively [16,17]. These events lead to chronic low grade inflammation and premature cell death by apoptosis or pyroptosis in DR.

Diabetes strongly induces TXNIP in the retina and causes retinal cell injury and early cell death [6-9]. TXNIP knockdown by siRNA prevents several retinal abnormalities seen in early DR such as neuronal injury, Muller cell activation (gliosis) and capillary basement membrane thickening [7,8]. Therefore, knocking down or out TXNIP in the retina via gene therapy methods will be one way to prevent or slow down the progression of DR [8,9]. To perform this, the TXNIP promoter may be linked to a redox protein or to a shRNA targeting of genes that causes oxidative stress and/or inflammation or to TXNIP itself as shown in Figure 1A. For gene delivery to the retina, adeno-associated viral vectors (AAVs) such as AAV2, AAV5, AAV2/8, AAV9 as well as other modified vectors can be used [16]. These vectors can be delivered into the retina via an intravitreal or subretinal injection. The retina being a closed organ is relative immune privileged and systemic immune response is minimal. In addition, small genetic materials are needed for therapy when compared to that needed for other organ therapy or systemic treatment. Therefore, retinal gene therapy is one potential method appropriate (cost effectiveness) for long-term treatment in chronic ocular diseases. Because of the blood-retinal barrier, drug treatment via the systemic route does not reach the retina while repeated injection of proteins, peptides, antibodies and drugs may further lead to other complications of the eye [18]. So far, most treatment options for advanced diabetic retinopathy treatments have been ineffective for most patients and are unsatisfactory. Therefore, development of new innovative and effective methods including gene therapy is warranted.

## Human Adipose Stem Cells

Among the stem cells accessible for autologous cell therapy are adipose tissue and bone marrow. Adipose tissue-derived stem cells (ASC) may be harvested from individuals via liposuction while bone marrow derived stems cells (BMSC) is not readily accessible for most patients [19]. Furthermore, inducible pluripotent cells (iPSC) may be generated from patient fibroblast [20] and used in gene and tissue engineering to produce insulin or a gene that induces insulin production. Several approaches have been attempted for pancreatic beta cell transplant and other cell therapy models including encapsulated cell transplant for Type 1 diabetes [21]. Nonetheless, successful clinical application is yet to be achieved. One obstacle is that beta cells do not survive long outside their pancreatic environment, and also due to immune attack, they require multiple transplants. In addition, heterologous transplant needs anti-rejection drugs to maintain the transplanted tissue. In this regard, treatment (and/or cure) of diabetes using TXNIP promoter linked with human insulin gene engineered into ASCs to make beta-like cells, which sense and produce insulin under high glucose is innovative and clinically applicable via subcutaneous transplant. Being autologous ASC cells they will not be rejected by the systemic immune response and adipose cells live longer in subcutaneous environment.

## Construction of TXNIP Promoter-Insulin Gene Vectors and Therapeutic Approaches

Vectors or plasmids are genetic carriers for gene expression in cells. We may construct a vector that contains a human TXNIP promoter, which is linked to a human insulin gene with or without PDX1, a transcription factor that is expressed in beta cells and require for insulin production and processing. Insulin is synthesized as a pro-insulin form and processed to give two peptides, namely insulin and C-peptide. So, cells secrete 1:1 ratio of insulin and C-peptide. Because insulin half-life is short, most investigators measure C-peptide in tissue culture media or blood plasma to determine equivalent insulin content. AAV2/8 and others (mentioned above), which are known to incorporate into a defined locus in chromosome 19 and has relatively low immunogenicity, may be used for gene transduction in ASC. Furthermore, lentivirus can also be utilized as it transduces non-dividing cells such as retinal cells. However, lentivirus will incorporate into the genome and may introduce unexpected genomic mutations. Once an appropriate construct is made, then they may be transduced or incorporated into ASC cells as shown in Figure 1B.

### A. Step 1. *In vitro studies*:

Optimization of the vector constructs and stable transfection into ASCs is required. After that, the engineered ASCs may be treated with normal glucose (5mM) or high glucose (25 mM) conditions in *in vitro* cultures for various time periods and examine both the level of insulin production inside the cells and secretion into the medium. Cellular oxidative stress under high glucose environment and their viability may also be monitored to ascertain cell health during the experimental period.

### B. Step 2. *In vivo studies:*

After the *in vitro* studies and a demonstration that insulin is synthesized and secreted by these ASC cells under high glucose environment, we may then proceed to the next in vivo studies using nude diabetic mice. In such a study, nude mice may be induced diabetes with streptozotocin (STZ), which destroys beta cells and generates a Type 1 diabetes model. Next, the TXNIP promoter insulin-gene engineered ASC may be transplanted subcutaneously in these diabetic nude mice to determine if they are able to maintain starving blood glucose levels when compared to untreated diabetic mice that received ASC cells without the insulin gene construct.

### C. Step 3. Human trials:

Subsequently, when the animal studies are successful, and a proof of concept is established, then the technology may be applied to human patients in later trials. The safety and long-term survival of these transplanted cells need to ascertained before going further to human applications.

## Conclusion

The idea that a TXNIP promoter construct could be used for gene and tissue engineering of adipose stem cells for the treatment of diabetes and its complications is innovative and will be practical if and when the proof of concept of this method is validated. Subcutaneous ASC is easily accessible from patents and it can be engineered ex-vivo, expanded, and transplanted subcutaneously in its own environment without provoking immune responses. In addition to diabetes, TXNIP is also known to be upregulated in age-related neurodegenerative diseases such as Alzhemer’s and Parkinson’s diseases and Amyotrophic lateral sclerosis (ALS) [22,23]. In these neuronal diseases, mitochondrial dysfunction, protein misfolding and mitophagy deregulation are known to be critically involved in pathogenesis. The TXNIP promoter linked with its own shRNA to knock itself down (Fire Fights Fire, F3 approach) or used with a redox protein such as Trx1, copper-zinc superoxide dismutase (Cu/Zn SOD1) or a neurotrophic factor (BDNF) may be linked to prevent mitochondrial dysfunction, bioenergetics deficiency and neuronal cell death [24,25]. The TXNIP promoter linked gene expression may also have certain advantages in application in other diseases in that such a construct will be activated by hyperglycemia encountered after each meal, which may be considered its physiological inducer. Recently, we applied a patent for this technology and further studies will be performed [26].

## Figures and Tables

**Figure 1:**
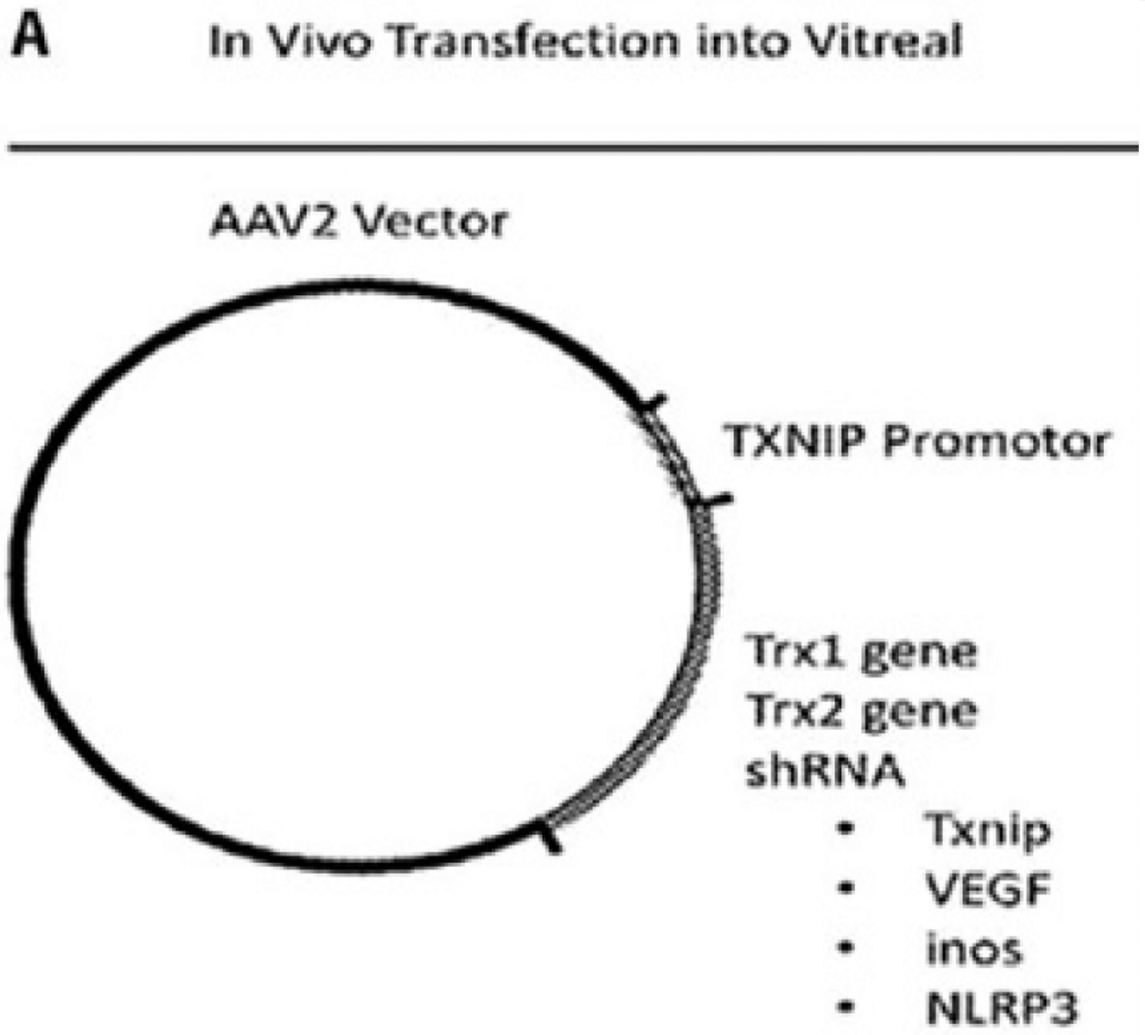
(A) Proposed Gene Constructs and Therapy Approaches: Steps for the construction of the TXNIP-promoter-gene vector for intravitreal delivery.

**Figure 1B:**
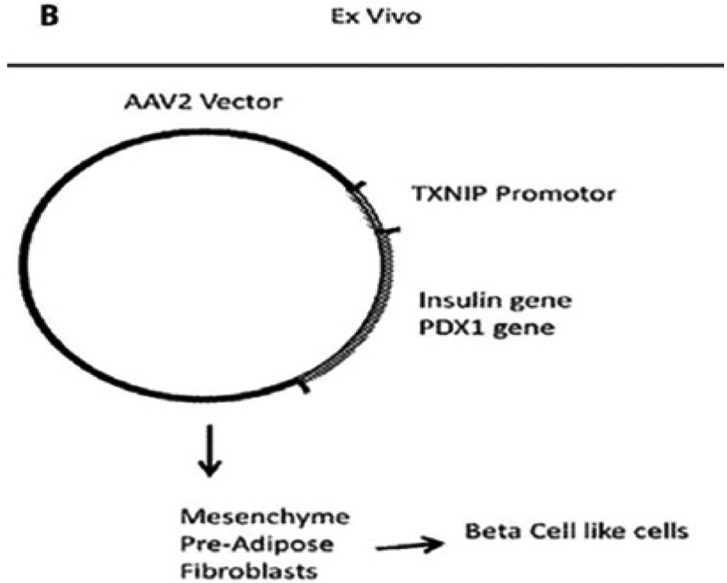
(B) For subsequent cell transplantation in diabetic animals *in vivo*, these constructs (or modified gene construct and their vectors) may be used for tissue engineering and further clinical applications for the treatment of Type 1/Type 2 diabetes and its complications including DR as well as neurodegenerative diseases [26].
